# The P300 acetyltransferase inhibitor C646 promotes membrane translocation of insulin receptor protein substrate and interaction with the insulin receptor

**DOI:** 10.1016/j.jbc.2022.101621

**Published:** 2022-01-21

**Authors:** Jinghua Peng, Balamurugan Ramatchandirin, Yu Wang, Alexia Pearah, Kopperuncholan Namachivayam, Risa M. Wolf, Kimberley Steele, Krishnan MohanKumar, Liqing Yu, Shaodong Guo, Morris F. White, Akhil Maheshwari, Ling He

**Affiliations:** 1 Department of Pediatrics, Johns Hopkins University School of Medicine, Baltimore, Maryland, USA; 2 Department of Surgery, Johns Hopkins University School of Medicine, Baltimore, Maryland, USA; 3 Division of Endocrinology, Diabetes, and Nutrition, University of Maryland School of Medicine, Baltimore, Maryland, USA; 4 Department of Nutrition and Food Science, Texas A&M University, College Station, Texas, USA; 5 Division of Endocrinology, Boston Children's Hospital, Boston, Massachusetts, USA; 6 Department of Pharmacology and Molecular Sciences, Johns Hopkins University School of Medicine, Baltimore, Maryland, USA

**Keywords:** inhibitor C646, insulin receptor, insulin receptor substrate, tyrosine phosphorylation, HFD, high-fat diet, IR, insulin receptor, IRβ, beta subunit of the insulin receptor, IRS, insulin receptor substrate, LPS, lipopolysaccharide, PH domain, pleckstrin-homology domain, PTB, phosphotyrosine binding, T1D, type 1 diabetes, T2D, type 2 diabetes

## Abstract

Inhibition of P300 acetyltransferase activity by specific inhibitor C646 has been shown to improve insulin signaling. However, the underlying molecular mechanism of this improvement remains unclear. In this study, we analyzed P300 levels of obese patients and found that they were significantly increased in liver hepatocytes. In addition, large amounts of P300 appeared in the cytoplasm. Inhibition of P300 acetyltransferase activity by C646 drastically increased tyrosine phosphorylation of the insulin receptor protein substrates (IRS1/2) without affecting the tyrosine phosphorylation of the beta subunit of the insulin receptor (IRβ) in hepatocytes in the absence of insulin. Since IRS1/2 requires membrane translocation and binding to inositol compounds for normal functions, we also examined the role of acetylation on binding to phosphatidylinositol(4,5)*P*2 and found that IRS1/2 acetylation by P300 reduced this binding. In contrast, we show that inhibition of IRS1/2 acetylation by C646 facilitates IRS1/2 membrane translocation. Intriguingly, we demonstrate that C646 activates IRβ′s tyrosine kinase activity and directly promotes IRβ interaction with IRS1/2, leading to the tyrosine phosphorylation of IRS1/2 and subsequent activation of insulin signaling even in the absence of insulin. In conclusion, these data reveal the unique effects of C646 in activating insulin signaling in patients with obesity and diabetes.

Obesity, which has reached alarming levels worldwide, is associated with an increased risk of metabolic abnormalities including type 2 diabetes (T2D). T2D accounts for more than 90% of diabetes cases, while type 1 diabetes (T1D) accounts for about 5 to 10% of diabetes. Insulin deficiency in T1D as well as insulin resistance and insufficient insulin secretion from impaired pancreatic β cells in T2D result in increased glucose production in the liver, which is the major cause of hyperglycemia in diabetic patients (1, 2, 3, 4). Several lines of evidence indicate that the impairment of proximal insulin signaling results in insulin resistance in obesity and T2D (5, 6).

Insulin binds to the extracellular α-subunit of the insulin receptor (IR), causing transphosphorylation of integral membrane β-subunit of IR by its intrinsic tyrosine kinase activity (7, 8). Phosphorylation of the IR at Y972 by insulin generates an NPE(p)Y_972_ motif that helps the recognition and binding of the phosphotyrosine binding (PTB) domain in the insulin receptor substrate (IRS) proteins to the IR (2, 9, 10). This selective and regulated binding of IRS to IR, even though with low affinity (11), adds an essential level of signaling specificity (12). IRS can bind to the plasma membrane through the pleckstrin-homology domain (PH domain), which can recognize and bind to membrane phospholipids or acidic peptide motifs in membrane proteins (13, 14, 15). Both the PH domain and PTB domain in IRS contribute to the membrane translocation and interaction of IRS to IR (16, 17), where the tyrosine residues in IRS can be phosphorylated by the tyrosine kinase activity of IRβ. This action plays a pivotal role in the activation of intracellular insulin signaling (18). The phosphorylation of tyrosine residues in IRS by the IR recruits p85 subunit in PI3K to IRS and the plasma membrane, then PI3K synthesizes PtdIns(3,4,5)*P*3 (PIP3) in cellular membranes and subsequent activation of downstream mediator AKT (2), resulting in the suppression of hepatic glucose production (19, 20, 21). Mice with liver-specific double IRS1 and IRS2 knockout exhibit severe hyperglycemia (5, 22), suggesting that hepatic IRS1 and IRS2 are the critical mediators of insulin’s regulation of glucose metabolism.

Obesity and metabolic disorders are associated with increased blood bacterial lipopolysaccharide (LPS) level and its initiated low-grade of inflammation (23), and activation of the deacetylase Sirtuin 1 restored insulin sensitivity in tissues with insulin resistance (24, 25, 26). We reported that endotoxin-induced acetyltransferase P300 can impair insulin signaling by acetylating IRS1 and 2; in contrast, acetyltransferase chemical inhibitor C646 improves insulin signaling (27). However, how inhibition of P300-mediated IRS acetylation by C646 improves insulin signaling remains unknown. In this study, we defined the molecular mechanism by which inhibitor C646 directly activates insulin signaling in hepatocytes of obesity and diabetes.

## Results

### Elevated P300 impairs insulin signaling in hepatocytes

**Figure 1:**
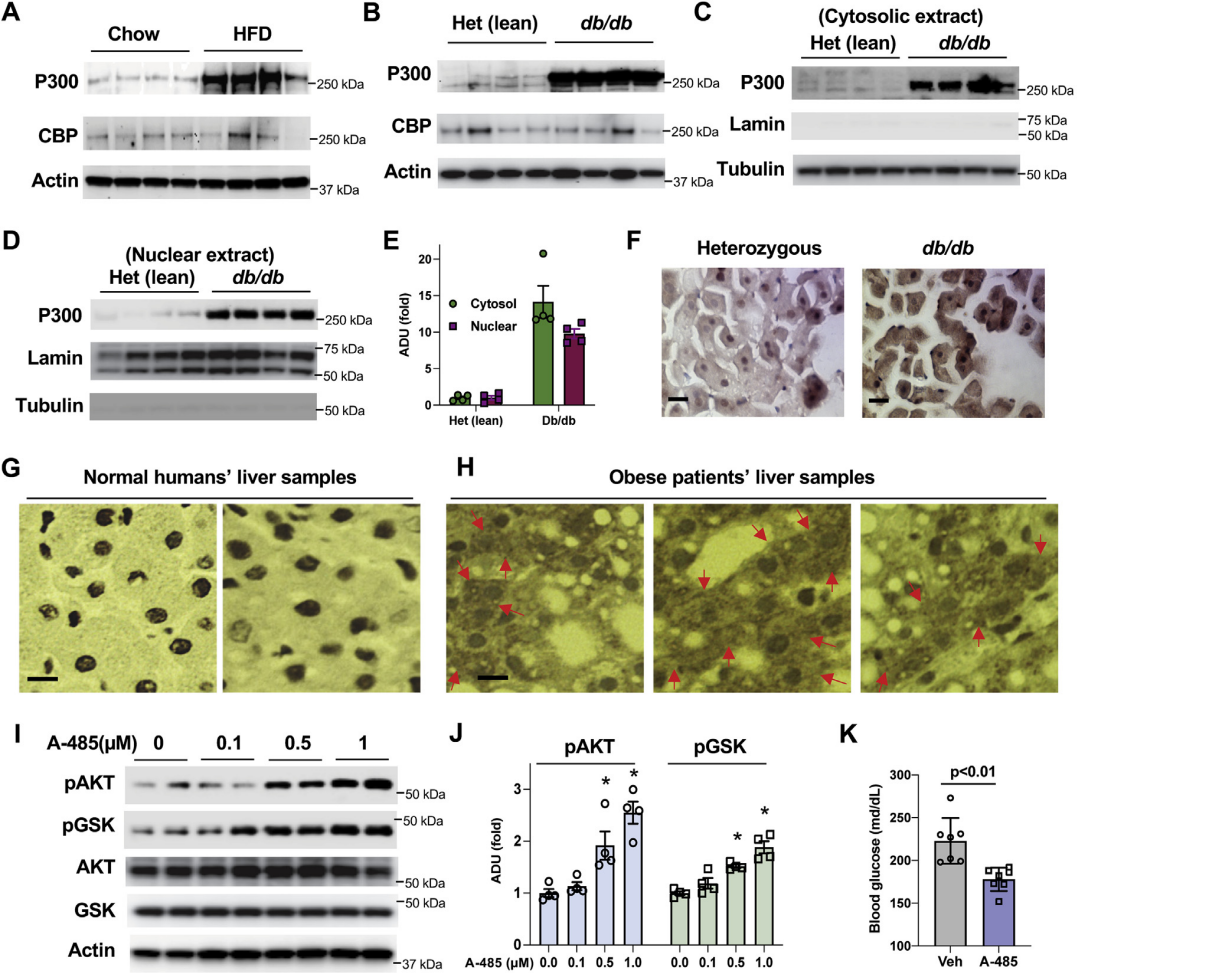
**Elevated P300 impairs insulin signaling in hepatocytes.***A*, C57BL6 mice were fed on a regular chow diet or high-fat-diet (HFD) for 4 weeks, liver tissues were collected (n = 4). *B*, the protein levels of P300 and CBP in the liver of age-matched heterozygous (Het) lean mice and *db/db mice* (n = 4). *C*–*E*, cytosolic (*C*) and nuclear (*D*) extracts were prepared from the liver of (Het) lean mice and *db/db* mice as in (*B*), and densitometric analysis of cytosolic and nuclear P300 protein levels (*E*) (n = 4). *F*, immunostaining of P300 in frozen liver tissues of heterozygous lean and *db*/*db mice*. *G* and *H*, paraffin-embedded liver samples from two normal humans and three obese patients were sectioned and immunostained with P300 antibody. Each panel represents an individual liver sample. Note P300 mostly inside the nucleus in normal humans’ hepatocytes (*G*), but large amounts of P300 appeared in the cytoplasm of liver hepatocytes of obese patients (*H*). *Red arrows* indicate cytoplasmic localization of P300. *I* and *J*, 48 h after the seeding, primary hepatocytes were cultured in DMEM with 5% FBS and treated with indicated amounts of A-485 for 3h (*I*), and densitometric analysis of pAKT and pGSK (*J*) (n = 4). *K*, C57BL6 mice were fed on an HFD for 2 weeks, then treated with vehicle or A-485 (15 nmol/g/day) *via* intraperitoneal injection for another 2 weeks. Fasting blood glucose levels (6 h) (n = 7). The y-axis has been broken and begins at 100 mg/dl. ∗*p* < 0.05, paired sample *t* test between groups. Scale bar, 10 μm. CBP, Creb-binding protein.

Our previous report showed that high-fat-diet (HFD) feeding led to increased P300 protein levels in the liver (27). We validated these findings in another animal study and found that HFD feeding significantly increased P300 protein levels in the liver (Fig. 1*A*). In genetically obese *db/db* mice, there are drastic increases in total, cytosolic, and nuclear P300 protein levels in the liver compared with age-matched heterozygous lean mice (Fig. 1, *B*–*E*). Consistently, immunostained liver sections by validated P300-specific antibody (27) showed increased amounts of P300 in the cytoplasm of liver hepatocytes of *db/db* mice (Fig. 1*F*). Next, we examined P300 protein levels in two normal human liver samples and three obese patients and found that obese patients had significantly elevated liver P300 protein levels and large amounts of P300 in the cytoplasm of hepatocytes, contrasting with the mostly nuclear localization of this protein in normal humans (Fig. 1, *G* and *H*). In agreement with our previous report (27), inhibition of the acetyltransferase activity by a potent inhibitor A-485 (inhibitor for both CBP and P300’s acetyltransferase) significantly increased AKT and GSK phosphorylation in primary hepatocytes prepared from HFD-fed mice (Fig. 1, *I* and *J*) and in Hepa1-6 cells (Fig. S1, *A* and *B*). In HFD-fed mice, treatment with A-485 for 2 weeks improved the hyperglycemia (Fig. 1*K*).

### P300 acetyltransferase-specific inhibitor C646 activates insulin signaling in the absence of insulin and FBS

**Figure 2:**
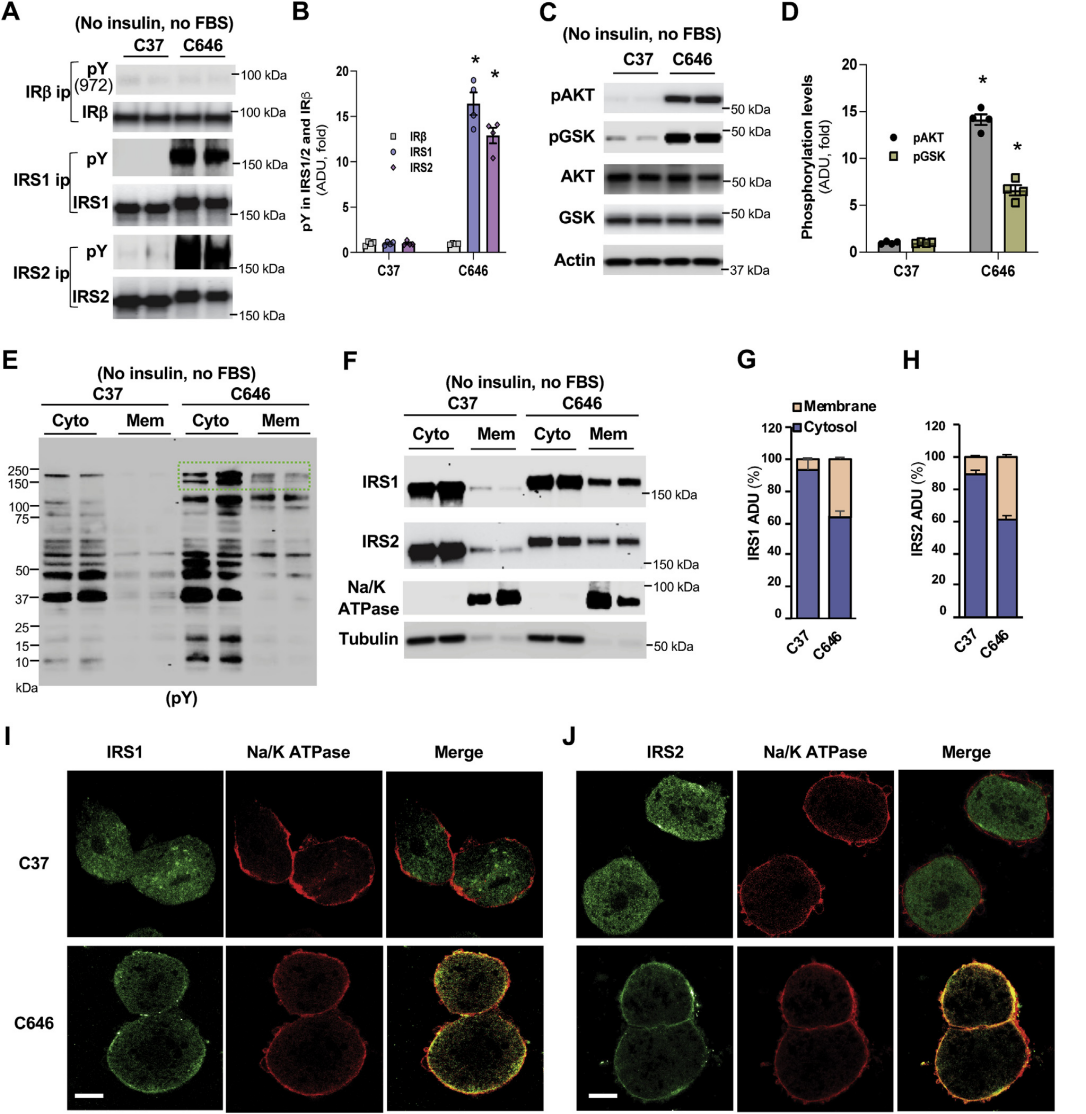
**C646 activates insulin signaling and increases IRS membrane translocation in the absence of insulin.***A*–*D*, 24 h after the seeding of Hepa1-6 cells, cells were washed with PBS, and then cultured in FBS-free medium with 20 μM of C37 or C646 for 4 h. Cell lysates were immunoprecipitated with indicated antibodies at 4 °C overnight (*A*), densitometric analysis of phospho-tyrosine in IRβ and IRS1 and 2 (*B*), the phosphorylation levels of AKT and GSK (*C*) and their densitometric analysis (*D*) (n = 4). *E*–*H*, Hepa1-6 cells were treated as above, cytosolic and membrane fractions were prepared and immunoblotted with phospho-tyrosine antibody (*E*), the protein levels of IRS1 and 2 were also determined (*F*) and their corresponding densitometric analysis (*G* and *H*) (n = 4). *I* and *J*, 24 h after the plating of primary hepatocytes prepared from HFD-fed mice, cells were washed, and cultured in FBS free medium 1 h (serum starvation), then treated with 20 μM of C37 or C646 for 4 h, followed by staining with specific antibodies against Na/K ATPase, IRS1 (*I*) or IRS2 (*J*). ∗*p* < 0.05, paired sample *t* test between groups. Scale bar, 10 μm. IRS, insulin receptor substrate.

In Hepa1-6 cells, there is a significant portion of P300 located in the cytoplasm (Fig. S2, *A* and *B*). To test the effects of P300 acetyltransferase activity on insulin signaling, we treated Hepa1-6 cells with P300 acetyltransferase-specific inhibitor C646 and control inactive compound C37 (28, 29) in the absence of both insulin and FBS and found that C646 treatment led to over tenfold increase in the tyrosine phosphorylation of IRS1 and 2 (Fig. 2, *A* and *B*), along with their mobility shift that may be caused by their tyrosine phosphorylation (Fig. S2, *C* and *D*). Treatment with C646 significantly augmented AKT and GSK phosphorylation (Fig. 2, *C* and *D*). However, the tyrosine phosphorylation at Y972 and total tyrosine phosphorylation of IRβ remained unchanged after treatment with C646 (Figs. 2*A* and S2, *E* and *F*), indicating that C646-mediated activation of insulin signaling is not through augmenting the binding of IRS1 and 2 to the NPE(p)Y_972_ motif in IRβ (16).

### C646 facilitates the membrane translocation of IRS1 and 2 in the absence of insulin

To determine how C646 activates cellular insulin signaling in the absence of insulin, we prepared cytosolic and membrane fractions from Hepa1-6 cells treated with control inactive compound C37, or inhibitor C646, in the absence of both insulin and FBS treatment because the membrane translocation of IRS1 and 2 is required for the activation of insulin signaling (2). As shown in Figure 2*E*, C646 treatment increased the total tyrosine phosphorylation levels in both cytosolic and membrane fractions (Figs. 2*E* and S2*G*). In both cytosolic and membrane fractions, C646 treatment increased the tyrosine phosphorylation levels in positions that correspond to the IRS1 and 2 proteins in the immunoblot (highlighted with the green square) (Fig. 2*E*). Of particular interest, C646 treatment significantly increased membrane-located IRS1 and 2 (6.9%–36.0%, IRS1; 11%–38.7%, IRS2) (Fig. 2, *F*–*H*). C646 treatment caused the mobility shift of IRS1 and 2 in both cytosolic and membrane fractions, suggesting that the tyrosine residues of IRS1 and 2 were phosphorylated in both cellular compartments. To further prove that C646 can facilitate the membrane translocation of IRS1 and 2, primary hepatocytes from HFD-fed mice were prepared and subjected to serum starvation for 1 h before the addition of inhibitor C646. Indeed, C646 treatment increased the membrane located IRS1 and 2 in the absence of insulin treatment (Figs. 2, *I* and *J* and S2, *H* and *I*).

### Blockade of P300 acetylation sites in IRS1 and 2 by K to R mutation increases IRS membrane localization

**Figure 3:**
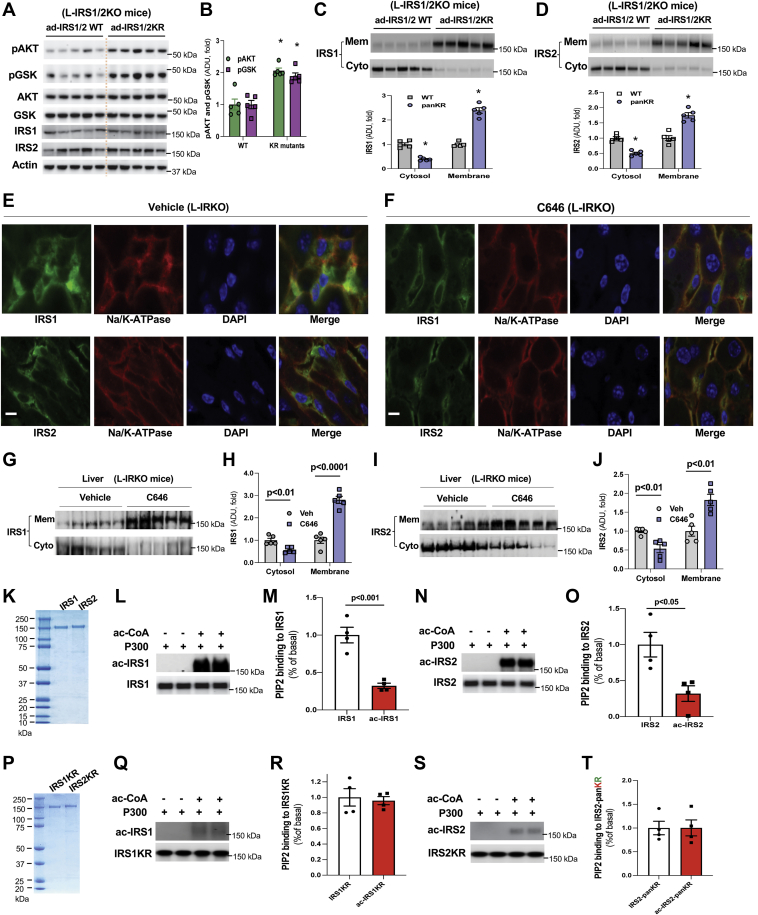
**Blockade of IRS acetylation facilitates their membrane translocation.***A*–*D*, homozygous double-floxed IRS1 and 2 mice were injected (jugular vein) with AAV8-TBG-Cre and adenoviral IRS1/2-WT, or adenoviral IRS1/2KR mutants, then mice were fed an HFD for 19 days, liver samples were collected. Phosphorylation levels of AKT and GSK in the liver (*A*) and their densitometric analysis (*B*) (n = 5). Cytosolic and membrane fractions were prepared from liver tissues and examined with anti-IRS1 antibody and densitometric analysis (*C*) or with anti-IRS2 antibody and densitometric analysis (*D*) (n = 5). *E*–*J*, HFD-fed (4 weeks) floxed IR mice were injected (jugular vein) with AAV8-TGB-Cre (1 × 10ˆ11 GC/mouse) to generate liver-specific IR knockout (L-IRKO) mice. After viral injection, mice were treated with vehicle or C646 (20 nmol/g/day) for 2 weeks. Liver samples were subjected to immunofluorescence staining, and images were acquired using confocal microscope (*E* and *F*). Cytosolic and membrane fractions were prepared from the liver tissues and examined with anti-IRS1 antibody (*G*) and densitometric analysis (*H*) or with anti-IRS2 antibody (*I*) and densitometric analysis (*J*) (n = 5). *K*, purified IRS1 and 2 proteins were subjected to SDS-polyacrylamide gel electrophoresis and stained with Colloidal blue. *L*–*O*, purified IRS1 and 2 proteins were incubated with 0.2 μg P300 protein at 37 °C for 2 h to acetylate IRS, then IRS1 or IRS2 antibody was added along with agarose bead and incubated 4 °C overnight to pull down IRS proteins, after washing, PtdIns(4,5)*P*2-fluorescein was added and incubated in the dark with rotation for 50 min. Unbound PtdIns(4,5)*P*2-fluorescein in the supernatant was removed after spin, and IRS bound PtdIns(4,5)*P*2-fluorescein was determined using plate reader (n = 4). *P*, purified IRS1 and 2 KR mutated proteins, in which all identified P300 acetylation lysine residues were substituted with arginine, were subjected to SDS-polyacrylamide gel electrophoresis and stained with Colloidal blue. *Q*–*T*, purified IRS1 and 2 KR mutated proteins were incubated with 0.2 μg P300 protein, then IRS1 or IRS2 antibody was added along with agarose bead to pull down IRS proteins, followed by incubation with PtdIns(4,5)*P*2-fluorescein to determine PIP2 binding to KR mutated IRS1 and 2 protein as above (n = 4). ∗*p* < 0.05, paired sample *t* test between groups. Scale bar, 10 μm. HFD, high-fat diet; IRS, insulin receptor substrate.

We found previously that seven lysine residues in IRS1 and 15 lysine residues in IRS2 can be acetylated and generated adenoviral expression vectors, in which these acetylated lysine residues in IRS1 and 2 were substituted with arginine, as mimics of nonacetylated lysine (KR mutants) (27). To examine whether the blocking of IRS1 and 2 acetylation by KR mutations can improve insulin signaling, we generated liver-specific IRS1 and IRS2 knockout mice by injection (jugular vein) of AAV8-TBG-Cre into double IRS1 and IRS2 floxed mice alone with the injection of adenoviral expression vectors containing IRS1 and 2 or their KR mutations (1 × 10^10^ GC/IRS/mouse). The protein levels of expressed wild-type IRS1 and 2, and their mutant proteins were comparable with their corresponding endogenous protein levels in the liver of HFD-fed mice. At 19 days after the viral injections, liver tissues were collected. There were significantly increased liver AKT and GSK phosphorylation in mice with the expression of mutated KR proteins of IRS1 and 2 compared with mice with the expression of wild-type IRS1 and 2 proteins (Fig. 3, *A* and *B*). Additionally, there were significantly higher levels of membrane located IRS1 and 2 proteins along with reduction of cytosolic IRS1 and 2 proteins in the liver of mice with the expression of mutated KR proteins of IRS1 and 2 (Fig. 3, *C* and *D*). These results verified that acetylation of IRS1 and 2 has a negative impact on their membrane translocation.

### C646 increases the membrane localization of IRS1 and 2 in the liver of HFD-fed mice without insulin receptor

C646 treatment activated insulin signaling and increased membrane located IRS1 and 2, while the tyrosine phosphorylation at Y972 and total tyrosine phosphorylation of IRβ remained unchanged (Figs. 2, *A*–*H* and S2, *E* and *F*). To test whether IR is required for C646 stimulated membrane translocation of IRS1 and 2, we generated liver-specific IR knockout (L-IRKO) mice by injection of AAV-TBG-Cre to HFD-fed floxed IR mice (Fig. S3*A*) and treated these mice with inhibitor C646 (20 nmol/g/day) *via* intraperitoneal injection for 2 weeks (29). In the liver of L-IRKO mice treated with vehicle control, predominant amounts of IRS1 and 2 were located in the cytoplasm (Fig. 3*E*); in contrast, C646 treatment facilitated IRS1 and 2 membrane localization (Fig. 3*F*). In cytosolic and membrane fractions prepared from these L-IRKO mice (Fig. S3*B*), C646 treatment significantly increased the protein levels of both IRS1 and 2 in the membrane fractions, while significantly reducing protein levels of both IRS1 and 2 in the cytosolic fractions compared with their protein levels of vehicle-treated L-IRKO mice (Fig. 3, *G*–*J*). These results indicate that IR is not required for C646 stimulated membrane translocation of IRS1 and 2. However, C646 was unable to improve insulin signaling in the liver of mice without IR (data not shown).

### IRS acetylation negatively affects their bindings to phosphatidylinositol(4,5)P2

Membrane translocation and binding to inositol compounds in the membrane can affect IRS′ function. PH domain in IRS can recognize and bind to membrane phospholipids (13, 14, 15). Based on the above data shown that C646 treatment increased the membrane located IRS1/2 in the liver of HFD-fed mice without IR (Fig. 3, *E*–*J*), we reasoned that IRS acetylation may have a negative impact on IRS binding to phosphatidylinositol(4,5)*P*2 (PIP2), which is also the substrate of immediate downstream mediator of PI3K. To test whether IRS acetylation can reduce their bindings to PIP2 in the membrane as a mechanism of the impairment of insulin signaling by P300, we conducted *in vitro* binding assays using acetylated IRS1 and 2. First, we used P300 to acetylate purified IRS1 and 2 proteins (Fig. 3*K*) in the presence or absence of acetyl-CoA (groups without the addition of acetyl-CoA served as controls) (Fig. 3, *L* and *N*). Second, specific antibodies against IRS1 and 2 were used to immunoprecipitate acetylated or nonacetylated IRS1 and 2 to remove P300. Then, PIP2-fluorescein was added to the immunoprecipitates, and IRS-bound PIP2 was determined. We found that IRS acetylation by P300 reduced ∼70% bindings of PIP2 (Fig. 3, *M* and *O*). Furthermore, the substitution of all identified acetylated lysine residues with arginine (pan KR mutation) in IRS1 or 2 abolished P300’s negative effect on the binding of PIP2 to IRS1 and 2 protein (Fig. 3, *Q*–*T*). These results suggest that IRS acetylation may inhibit their membrane translocation through reduction of binding to PIP2. Since the acetylation of IRS1 and 2 could not affect their tyrosine phosphorylation by IRβ (Fig. S3, *C* and *D*); therefore, P300 impairment of insulin signaling is mainly through the prevention of the membrane translocation of IRS1 and 2.

### C646 directly promotes IRS binding to unphosphorylated IR*β*

**Figure 4:**
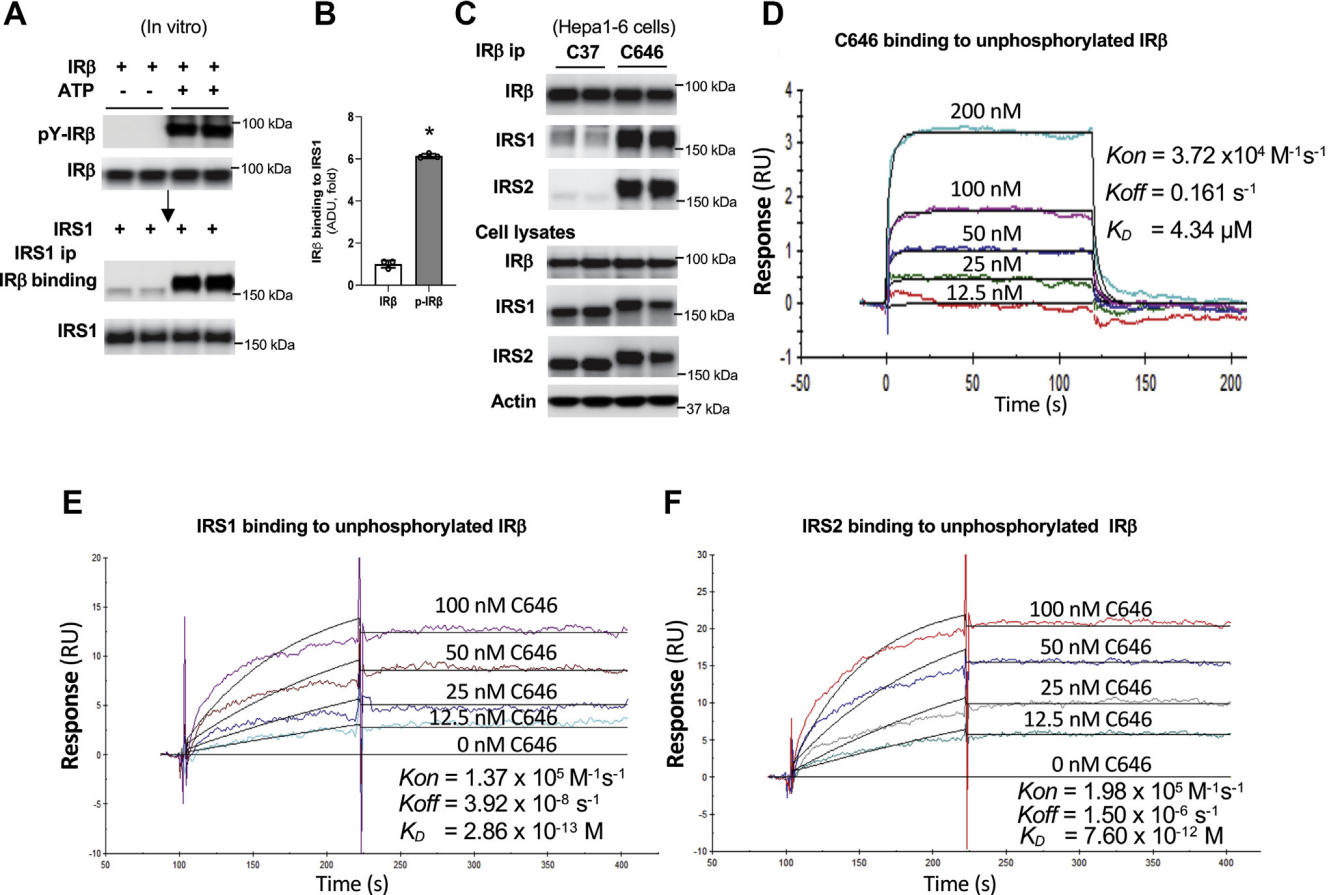
**C646 increases IRS′ bindings to unphosphorylated IRβ.***A* and *B*, unphosphorylated IRβ was incubated at 37 °C for 1 h in the absence or presence of ATP. Phosphorylated or unphosphorylated IRβ was incubated with IRS1 for 2 h, then IRS1-specific antibody was used to pull-down IRS1-associated IRβ at 4 °C overnight (*A*) and densitometric analysis of IRS1-associated IRβ (*B*) (n = 3). *C*, Hepa1-6 cells were treated as Figure 2*A*, IRβ-specific antibody was used to immunoprecipitate IRβ and associated proteins. *D*, unphosphorylated IRβ (ligand) was immobilized on the surface of a sensor chip, and different concentrations of C646 were injected and the bindings of C646 to IRβ were recorded in Biacore T200. *E* and *F*, unphosphorylated IRβ (ligand) was immobilized on the surface of a sensor chip, same amounts of either IRS1 (*E*) or IRS2 (*F*) together with indicated concentrations of C646 were injected. *K*_*on*_, the association rate constant; *K*_*off*_, the dissociation rate constant; the binding affinity *K*_*D*_ equals to *K*_*off*_/*K*_*on*_. IRβ, beta subunit of insulin receptor; IRS, insulin receptor substrate.

The tyrosine phosphorylation of IRβ at Y972 generates the NPE(p)Y_972_ motif that helps the recognition and binding of the PTB domain of IRS to the IR (2, 9, 10). We validated these findings and found that tyrosine phosphorylation of IRβ led to over fivefold more bindings of IRS1 and 2 to IRβ (Figs. 4, *A* and *B* and S4, *A* and *B*). However, in Hepa1-6 cells, C646 treatment did not increase the tyrosine phosphorylation at Y972 and total tyrosine phosphorylation of IRβ in the absence of insulin (Figs. 2, *A* and *B* and S2, *E* and *F*), but C646 treatment drastically increased the binding of IRS1 and 2 to IRβ (Fig. 4*C*). We reasoned that C646 may bind directly to IRβ, or IRS1 and 2, therefore, facilitating the interaction of IRβ with IRS1 and 2. To test this notion, we conducted Surface Plasmon Resonance (SPR) to determine whether C646 can bind to IRS1 and 2 or IRβ. We found that C646 can bind directly to unphosphorylated IRβ, but not to IRS1 and 2 (Figs. 4*D* and S4, *C* and *D*), and C646 binds to IRβ with relatively high affinity (*K*_*D*_ = 4.34 μM). Due to the fact that C646 can bind directly to unphosphorylated IRβ, we tested whether C646 has any effect on the binding of unphosphorylated IRβ with IRS1 or 2 using SPR. Strikingly, unphosphorylated IRβ can bind to either IRS1 or IRS2 with extremely high affinity (*K*_*D*_, 2.86 × 10^−13^ for IRS1; *K*_*D*_, 7.6 × 10^−12^ for IRS2) in the presence of C646 at nM concentrations (Fig. 4, *E* and *F*). These data are consistent with the findings that C646 treatment drastically increased the association of IRS1 and 2 with unphosphorylated IRβ in Hepa1-6 cells (Figs. 2, *A* and *B*, 4*C* and S2, *E* and *F*). Thus, in the absence of tyrosine phosphorylation of IRβ by insulin, it is the C646 that directly promotes IRβ bindings to IRS.

### C646 activates IR*β*′s tyrosine kinase activity

**Figure 5:**
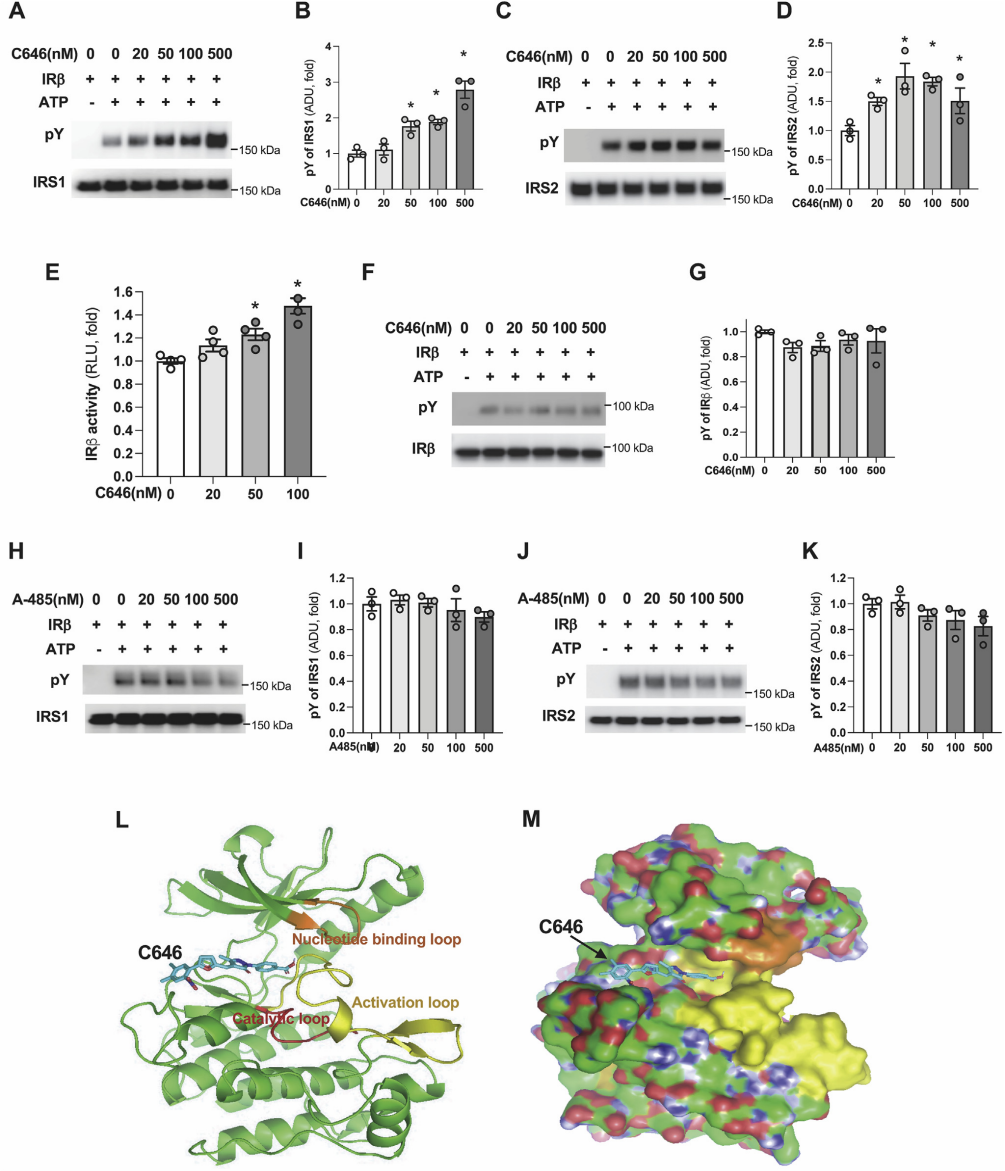
**C646 increases IRβ kinase activity.***A*–*D*, IRS1 plus IRβ (*A*) or IRS2 plus IRβ (*C*) were incubated with indicated concentrations of C646 at 4 °C for 1 h, then ATP was added to allow IRβ to phosphorylate IRS1 and 2 (30 °C, 15 min). Densitometric analysis of tyrosine phosphorylation of IRS1 (*B*) and IRS2 (*D*) (n = 3). *E*, indicated concentrations of C646 were added in the reaction containing 1 μg of insulin receptor, substrate peptide, and ATP, the kinase activity was determined by measuring the ADP production as described in Experimental procedures (n = 4). *F* and *G*, IRβ was incubated different concentration of C646 as in (*A*), ATP was added to allow IRβ to phosphorylate itself (30 °C, 15 min) (*F*) and densitometric analysis of tyrosine phosphorylation of IRβ (n = 3) (*G*). *H*–*K*, IRβ and IRS1 and 2 were incubated with indicated concentrations of A485 at 4 °C for 1 h, then ATP was added to allow IRβ to phosphorylate IRS1 (*H*) or IRS2 (*J*) (30 °C, 15 min) and densitometric analysis of tyrosine phosphorylation of IRS1 (*I*) and IRS2 (*K*) (n = 3). ∗*p* < 0.05, paired sample *t* test between groups. *L* and *M*, AutoDock Vina was used to predict binding information of C646 to IRK, and PyMOL was used to generate images of C646’s docking on the IRK in ribbon diagram (*L*) or surface presentation (*M*). Nucleotide binding loop in *orange*, catalytic loop in *red*, and activation loop in *yellow*. IRβ, beta subunit of insulin receptor; IRK, insulin receptor kinase; IRS, insulin receptor substrate.

To determine whether C646 promoting the binding of IRβ to IRS has any effect on the tyrosine phosphorylation of IRS by IRβ, we conducted *in vitro* phosphorylation assays, in which IRβ and IRS1 or IRS2 were incubated with different concentrations of C646, followed by addition of ATP to initiate the phosphorylation of IRS. We found that C646 significantly increased the tyrosine phosphorylation of both IRS1 and 2 by IRβ (Fig. 5, *A*–*D*). Having seen these data, we asked whether C646 can affect the enzymatic activity of IRβ. To test this hypothesis, we incubated the insulin receptor with different concentrations of C646, then determined its kinase activity on the phosphorylation of tyrosine residue in peptide substrate by measuring the production of ADP from ATP using InsR kinase assay. C646 did increase the kinase activity of insulin receptor by 50% at 100 nM concentration (Fig. 5*E*). The above data show that C646 not only promotes the binding of IRβ to IRS, but also activates the enzymatic activity of IRβ, leading to drastically increased bindings of IRβ to IRS1 and 2 (Fig. 4*C*) and tyrosine phosphorylation of IRS1 and 2 (Fig. 2, *A* and *B*).

However, interestingly, C646 had no effect on the tyrosine phosphorylation of IRβ itself (Fig. 5, *F* and *G*), suggesting that C646 cannot affect the transphosphorylation of IRβ. These results are consistent with the findings that C646 treatment could not change the tyrosine phosphorylation at Y972 and total tyrosine phosphorylation of IRβ (Figs. 2, *A* and *B* and S2, *E* and *F*). Another potent CBP and P300’s acetyltransferase inhibitor, A-485, was unable to affect the tyrosine phosphorylation of IRS1 and 2 by IRβ (Fig. 5, *H*–*K*). These findings may explain why C646 has a stronger effect than A-485 on the activation of the insulin signaling pathway (compared Figs. 1 with 2, *C* and *D*).

Since the crystal structure of unphosphorylated and low activity form of the insulin receptor kinase domain (IRK) have been determined (30), we used computational docking (AutoDock Vina) (31) to predict binding information of C646 to the IRK. The best binding mode with affinity value -7.8 (kcal/m) reveals that C646 may bind to the cleft between N- and C-terminal lobes, where both ATP and protein substrate-binding sites are located with the activation lobe also located nearby (Fig. 5, *L* and *M*). It is possible that the cleft-sitting C646 can cause conformational changes carried out by insulin-mediated autophosphorylation of tyrosine residues in IRβ, allowing IRβ binding to IRS with high affinity and also increasing the enzymatic activity through reduction of activation energy.

### C646 treatment improves insulin signaling in obesity

**Figure 6:**
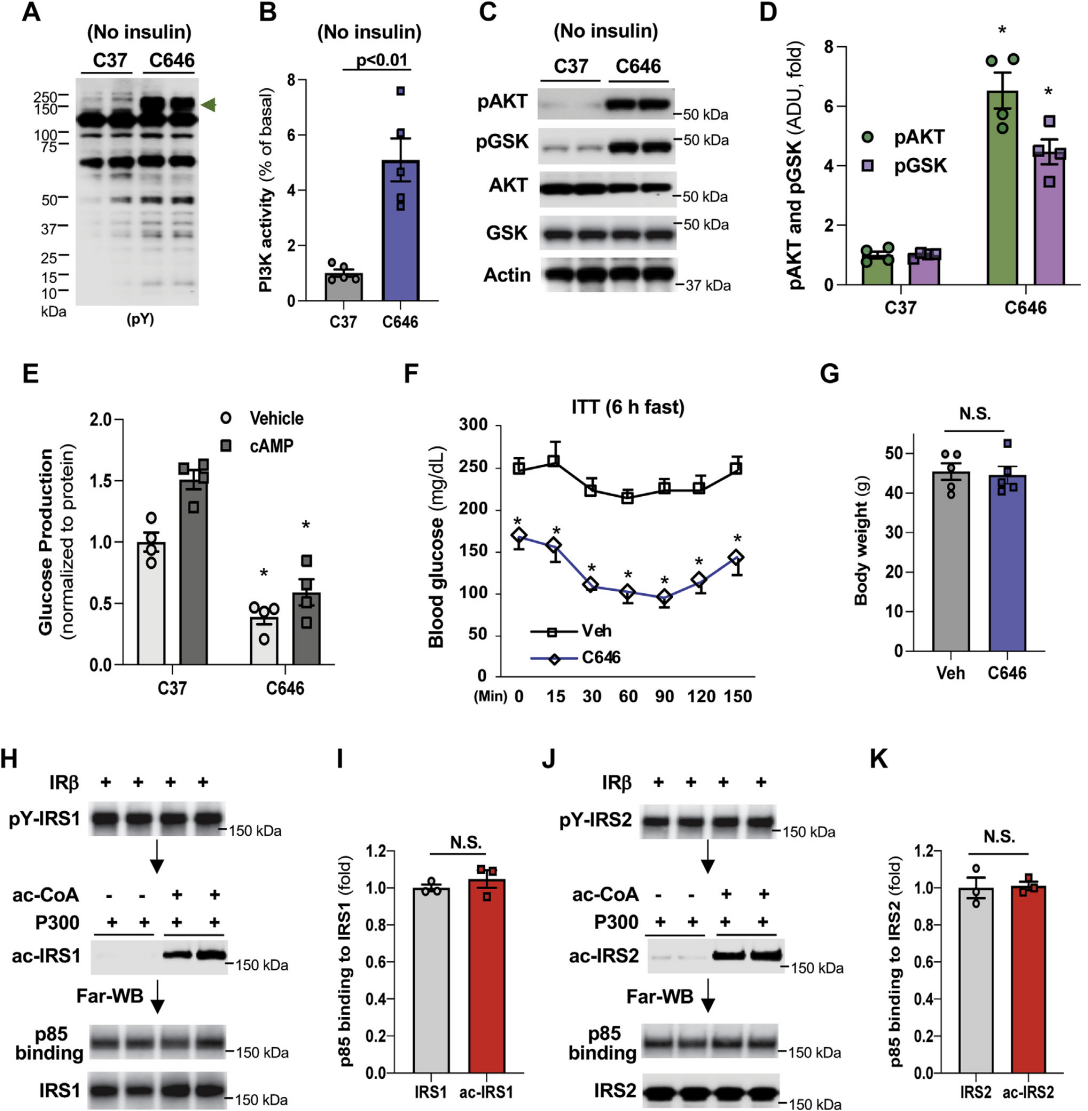
**C646 treatment improves insulin signaling in obesity.***A*–*D*, 24 h after the seeding, primary hepatocytes from *ob/ob* mice were subjected to serum starvation for 2 h, then treated with 20 μM of C37 or C646 for 3 h. The levels of phospho-tyrosine (*A*), PI3K activity (*B*), AKT and GSK phosphorylation (*C*) were determined, and densitometric analyses AKT and GSK phosphorylation (*D*) (n = 4∼5). *E*, after 48 h of seeding, primary hepatocytes were treated with 20 μM of C37 or C646 during serum starvation (3 h), washed with PBS, then incubated with 0.2 mM cAMP and 20 μM of C37 or C646 in the glucose production medium for anther 3 h (=4). *F* and *G*, obese *ob/ob* mice were treated with either vehicle or C646 (30 nM/g/day) for 10 days, insulin tolerance test was conducted (6 h fast, 0.8 u/kg) (n = 5) (*F*), and the body weight (*G*). *H*–*K*, purified IRS1 and 2 proteins were incubated with 0.2 μg of IRβ at 37 °C for 1 h, then 0.2 μg of P300 protein was added (37 °C, 2 h) to acetylate IRS, followed by Far-Western blot to examine the binding of IRS1 (*H*) and IRS2 (*J*) to p85, and their densitometric analyses (*I*–*K*) (n = 3). ∗*p* < 0.05, paired sample *t* test between groups. IRβ, beta subunit of insulin receptor; IRS, insulin receptor substrate.

We further tested C646’s effect on insulin signaling in hepatocytes from mice with obesity and diabetes. We prepared primary hepatocytes from obese *ob/ob* mice and treated the primary hepatocytes with control inactive compound C37 or inhibitor C646 after serum starvation. We found that, in the absence of insulin, C646 treatment drastically increased the tyrosine phosphorylation levels in positions that correspond to IRS1 and 2 proteins in the immunoblot (Fig. 6*A*). These data are consistent with the results that C646 treatment increased IRS tyrosine phosphorylation in Hepa1-6 cells (Fig. 2, *A* and *E*). C646 treatment also significantly increased PI3K activity and the phosphorylation of AKT and GSK in these primary hepatocytes in the absence of insulin (Fig. 6, *B*–*D*) and significantly suppressed glucose production (Fig. 6*E*). Moreover, in obese *ob/ob* mice, C646 treatment through intraperitoneal injection (30 nmol/g/day) for 10 days significantly improved insulin sensitivity and hyperglycemia without significant changes in body weight (Fig. 6, *F* and *G*). The above results show that the augmentation of ΙRβ activity, binding to IRS, and subsequent IRS tyrosine phosphorylation by C646 leads to the activation of PI3K-AKT signaling and the suppression of glucose production in the liver and alleviation of hyperglycemia.

### IRS acetylation has no apparent effects on the docking of downstream mediator p85 in PI3K

The phosphorylation of tyrosine residues in IRS1 and 2 by IRβ will generate pYMXM motifs and lead to the docking of p85 in PI3K to the membrane located IRS proteins and activation of downstream mediators in the PI3K-AKT signaling. We therefore determined whether IRS acetylation could affect the docking of p85 protein in PI3K to IRS1 and 2 proteins. First, we used IRβ to phosphorylate the tyrosine residues in IRS1 and 2, then P300 protein was added in the absence or presence of acetyl-CoA, followed by Far-Western blot to examine the docking of p85 protein to IRS1 and 2. We found that the acetylation of either IRS1 or IRS2 had no effects on the docking of p85 to these proteins (Fig. 6, *H*–*K*). Also, considering that the acetylation of IRS1 and 2 by P300 had no impact on the total tyrosine phosphorylation of these proteins, these data suggest that the interaction of p85 in PI3K with IRS proteins is not the main C646 targeting site. However, it is possible that the acetylation of IRS1 and 2 may have a negative effect on the phosphorylation of tyrosine residue in the YMXM motifs, thus affecting the binding of p85 to IRS.

## Discussion

Increased bacterial LPS leakage from the gut into circulation and its initiated low-grade of inflammation play important roles in the development of insulin resistance in obesity and T2D (3, 32, 33, 34). We found that LPS can induce P300 in hepatocytes *via* reduction of the ubiquitination and degradation of P300, and elevated P300 impairs insulin signaling by acetylating IRS1 and 2 (27, 35). Of note, P300 is a big protein; however, this protein has a relatively short half-life (5 h) (36), indicating that continuous protein translation in the cytoplasm is required to maintain the cellular P300 protein levels. Thus, P300 expression is readily regulated. Inhibition of P300 acetyltransferase activity by inhibitor C646 decreased IRS acetylation and improved insulin signaling in the liver. However, the molecular mechanisms by which there is negative impact of insulin signaling by P300-mediated IRS acetylation and improvement of insulin signaling by inhibitor C646 remain unclear. Insulin-resistant animals have decreased tyrosine phosphorylation of IRS in the liver (37); this can be caused by either impaired activation of IRβ intrinsic tyrosine kinase activity or decreased membrane translocation of IRS. Since insulin resistance occurs prior to β cell dysfunction (2), thus we tested the latter possibility and found that blocking IRS1 and 2 acetylation by substitution of all identified acetylated lysine residues with arginine as mimics of nonacetylated lysine significantly increased membrane located IRS1 and 2 proteins in the liver of HFD-fed mice. In addition, inhibition of P300 acetyltransferase by inhibitor C646 facilitated IRS1 and 2 membrane translocation in cultured hepatocytes. Since IRS can bind to phosphatidylinositols in the membrane *via* their PH domain and target IRS to IR (15, 38), we found that IRS acetylation drastically reduced their binding to PIP2. These data indicate that P300-mediated acetylation of IRS1 and 2 may impair insulin signaling by blocking IRS membrane translocation through reduction of their bindings to phosphatidylinositol. However, it is possible that IRS acetylation may affect their bindings to membrane-located protein as well. Consistent with the notion that the blockade of IRS acetylation augments their membrane translocation, we found that in HFD-fed L-IRKO mice, C646 can increase membrane located IRS1 and 2 proteins. These results also suggest that IR is not required for C646-facilitated IRS membrane translocation. Nevertheless, C646 could not activate insulin signaling in hepatocytes without insulin receptor.

Of particular interest, treatment with P300 acetyltransferase-specific inhibitor C646 is able to activate insulin signaling in the absence of insulin and FBS in hepatocytes, resulting in more than tenfold increase in the tyrosine phosphorylation of IRS1 and 2 and the activation of downstream mediators, such as PI3K, AKT and GSK. However, the tyrosine phosphorylation at Y972 and total tyrosine phosphorylation of IRβ were not affected by C646. The tyrosine phosphorylation of IRβ by insulin significantly increases its association with downstream mediator IRS; however, under the conditions of a lack of insulin and tyrosine phosphorylation of IRβ, it is C646 that binds directly to unphosphorylated IRβ to increase the affinity of IRβ to IRS1 and 2. Importantly, C646 can activate the tyrosine kinase activity of IRβ. Collectively, our data show that inhibition of IRS acetylation by C646 facilitates IRS1 and 2 membrane translocation and that the binding of C646 to IRβ increases IRβ′s affinity to IRS. Subsequently, C646-activated IRβ phosphorylates IRS1 and 2, leading to the activation of PI3K-AKT signaling. Additionally, tyrosine phosphorylation of IRS1 and 2 causes their mobility shift in immunoblots. We found the mobility shift of cytosolic IRS1 and 2 in C646-treated hepatocytes, which suggests that these IRS proteins are also phosphorylated at tyrosine residues. These IRS may direct PI3K to cellular membranes other than the plasma membrane to generate PIP3. Even though IRS acetylation has no effect on tyrosine phosphorylation by IRβ, IRS acetylation may reduce their bindings to the insulin receptor along with reduction of binding to PIP2, causing reduction of recruitment of p85-PI3K to the plasma membrane.

In T1D and frank T2D, the impairment of β cells will reduce the secretion of insulin into circulation, thus tyrosine phosphorylation at Y972 and total tyrosine phosphorylation of IRβ will be compromised. A decrease in generated NPE(p)Y_972_ motif will lead to reduction of IRS binding to IRβ and impairment of insulin signaling. Every component in the insulin signaling pathway does not miss even with their fluctuating protein levels, inhibitor C646 is able to activate insulin signaling in the absence of insulin. Therefore, in T1D, C646 treatment should activate insulin signaling through increasing the tyrosine kinase activity of IRβ and promoting the affinity of IRβ to IRS, leading to the activation of insulin signaling and suppression of liver glucose production. In T2D, besides the activation of the tyrosine kinase activity of IRβ and augmentation of the affinity of IRβ to IRS, inhibition of P300 acetyltransferase activity by C646 will facilitate IRS membrane translocation and make IRS available to IRβ. Thus, C646 is a potential agent to treat both T1D and T2D. Overall, this study provides new insight into the activation of cellular insulin signaling through stimulating IRS membrane translocation and association with IR and reveals unique effects of C646 on the activation of insulin signaling and improvement of insulin sensitivity. However, the binding sites of C646 on IRβ still need to be characterized.

## Experimental procedures

### Adenoviruses and adeno-associated viruses (AAV)

The mouse IRS1 and 2 genes were gifts from Ronald Kahn (Addgene plasmid # 11026, #11372) (39), and these IRS1 and 2 genes were used to generate FLAG-tagged IRS1 and 2. IRS1 and 2 mutants were created using site-directed mutagenesis (Stratagene) (40). FLAG-tagged IRS1-WT and -KR mutants and IRS2-WT and -KR mutants were subcloned into the pENTR2B vector (Invitrogen) and transferred into the pAd/CMV/V5-DEST vector (Invitrogen) by recombination to generate adenoviral expression clones as we described previously (27, 41).

### Glucose production assay

Mouse primary hepatocytes were cultured in William’s medium E supplemented with ITS (BD Biosciences) and dexamethasone. After 24 to 48 h of seeding, cells were washed with PBS twice, and the medium was changed to FBS-free DMEM supplemented with 20 μM of either C37 or C646. After 3 h of serum starvation, cells were washed twice with PBS, and 1 ml glucose production medium was supplemented with 20 μM of either C37 or C646. After 3 h incubation with glucose production medium, both the medium and cells were collected. The medium was used to determine glucose concentrations with EnzyChrom Glucose Assay Kit (29, 42, 43).

### Phosphorylation and acetylation assays

In phosphorylation assays, purified IRS1 and 2 proteins were incubated with 0.2 μg of IRβ (INSR-5093H, Creative Biomart) in reaction containing 25 mM Tris-HCl (pH7.5), 5 mM β-glycerophosphate, 2 mM DTT, 0.1 mM Na_3_VO_4_, and 10 mM MgCl_2_ at 30 °C for 15 min to 2 h in the absence or presence of 0.2 mM ATP. To acetylate IRS1 and 2, 2 μg of IRS1 or 2 was added to the reaction containing 50 mM Tris-HCl, pH 8.0, 5% glycerol, 0.1 mM EDTA, 50 mM KCl, 1 mM DTT, 1 mM PMSF, 10 mM sodium butyrate, 0.2 μg acetyl-CoA, and 1.5 μg P300 (Active Motif). Samples were incubated at 37 °C for 2 h.

### Measurement of PI3K activity

Twenty-four hours after the seeding, primary hepatocytes from *ob/ob* mice were subjected to serum starvation for 2 h, then treated with 20 μM of C37 or C646 for 3 h. P110α antibody (Cell signaling) was used to pull down PI3K, and its enzymatic activity was measured using the PI3K-Kinase Activity ELISA: Pico kit (Echelon Biosciences).

### Animal experiments

All animal protocols were approved by the Institutional Animal Care and Use Committee of the Johns Hopkins University. Male floxed homozygous IR mice, *db/db* mice, and *ob/ob* mice were purchased from the Jackson Laboratory. Floxed homozygous IR mice were fed on HFD (60% calories from fat) for 4 weeks, and AAV8-TGB-Cre (1 × 10ˆ11 GC/mouse) was injected *via* the jugular vein to generate liver-specific IR knockout mice. After viral injection, mice were treated with vehicle or C646 (20 nmol/g/day) for 2 weeks. To test C646’s effect on insulin sensitivity in obese *ob/ob* mice, 4-month-old *ob/ob* mice were randomly divided into two groups for which vehicle or C646 (30 nmol/g/day) was given through intraperitoneal injection for 10 days. Insulin tolerance test was performed after 6 h of fasting (0.8 unit/kg insulin). To test the effect of IRS1/2 mutants on their membrane translocation, 3-month-old homozygous double-floxed IRS1 and 2 mice were injected with adenovirus (1 × 10^10^ GC/mouse) through the jugular vein alone with AAV8-TGB-Cre (1 × 10ˆ11 GC/mouse). Two days after the viral injection, mice were placed on an HFD. After 19 days of HFD feeding, liver tissues were collected.

### Collection of human liver tissues

Liver tissues were obtained from the left lateral lobe of the liver of three obese patients (BMI, 37.17, 43.97, and 52.83, respectively) and immediately frozen on dry ice and stored at −80 °C. Normal human liver tissues were obtained through the Liver Tissue Cell Distribution System, which was funded by National Institutes of Health contract no. HHSN276201200017C (44).

### Determination of C646’s effect on the kinase activity of insulin receptor

We firstly employed different amounts (0.5, 1, 1.5, and 2 μg) of insulin receptor to determine the enzymatic activity needed in the InsR Kinase Assay (Promega) and found a linear curve of ADP production (consumption of ATP by insulin receptor). Subsequently, 1 μg of the insulin receptor was used in a 25 ul reaction containing different concentrations of C646, 0.2 μg of substrate peptide, 50 μM of ATP, and 1 × provided reaction buffer. The reactions were incubated at room temperature for 60 min. ADP-Glo Kinase Assay (Promega) was used to determine the ADP levels generated by the insulin receptor. Twenty-five microliters of ADP-Glo reagent was added to deplete residual ATP (room temperature, 40 min), then 50 ul of Kinase Detection Reagent was added to determine the ATP levels converted from ADP followed by the measurement of luminescence in BioTek Synergy H1 plate reader.

### Immunoprecipitation and preparation of cytosolic, membrane, and nuclear extracts

Cellular lysates were passed 15 times through a syringe needle to break cells on ice. ΙRβ, IRS1 and 2 were immunoprecipitated using ΙRβ antibody (Cell Signaling) and IRS1 and 2 antibodies (Millipore). The reaction was incubated at 4 °C for 16 h, followed by the addition of protein G beads (Active Motif) to pull down the target protein and its associated proteins. Cytosolic and nuclear extracts were prepared using the CelLytic NuCLEAR Extraction kit (Sigma). Membrane, cytosolic, and nuclear fractions were prepared from Hepa1-6 cells using Subcellular Protein Fraction Kit for Cultured Cells (Thermo Fisher) following manufacturer’s instruction.

### Far-Western blot

A far-Western blot was conducted using a modified method of Wu *et al.* (45). Purified IRS1 and 2 proteins were separated using a NuPAGE Novex Tris-Acetate Protein Gel (Thermo Fisher Scientific) and transferred onto a polyvinylidene fluoride membrane. The membrane was incubated in AC buffer (100 mM NaCl, 20 mM Tris (pH 7.6), 0.5 mM EDTA, 10% glycerol, 0.1% Tween-20, 2% skim milk powder, and 1 mM DTT) with 6 M guanidine–HCl for 30 min at room temperature. After washing, the membrane was blocked with 5% milk in PBST buffer (4 mM KH_2_PO_4_, 16 mM Na_2_HPO_4_, 115 mM NaCl (pH 7.4), and 0.05% Tween-20) for 1 h at room temperature. Subsequently, the membrane was incubated in protein-binding buffer (100 mM NaCl, 20 mM Tris (pH 7.6), 0.5 mM EDTA, 10% glycerol, 0.1% Tween-20, 2% skim milk powder, and 1 mM DTT, prepared freshly) plus 4 μg of p85 (16 h, 4 °C). After three washes with PBST buffer, the membrane was incubated with anti-p85 (Cell Signaling) at 4 °C overnight.

### Immunohistochemistry

Frozen liver samples of heterozygous lean and homozygous *db/db* mice were fixed with 4% paraformaldehyde in PBS for overnight at room temperature, then incubated in 30% sucrose overnight and frozen in OCT. Tissues were sectioned. After antigen retrieval using a microwave, sections were permeabilized with 0.1 Triton X-100 (10 min) and blocked in 3% horse serum for 1 h at room temperature. Paraffin-embedded human liver samples were sectioned. Sections were incubated with P300-specific antibody (ab14984, abcam) at 1: 20–200 dilution at 4 °C overnight.

### Confocal microscopy analysis

Liver tissues were harvested, washed with cold PBS, and fixed immediately with 4% paraformaldehyde in PBS for 6 h at 4 °C, then incubated in 30% sucrose overnight, and frozen in OCT. The frozen sections were cut at 10 uM thickness. Sections were washed in PBS, blocked, and permeabilized in blocking buffer (5% goat serum with 0.3% TritonX-100 in PBS) for 1 h at room temperature. For primary hepatocytes, 24 h after the seeding of primary hepatocytes, cells were washed with PBS and cultured in FBS-free DMEM for 1 h, then C37 or C646 was added. 4 h later, medium was removed. Cells were fixed with 4% paraformaldehyde in PBS, permeabilized with 1% Triton X-100, and blocked with 3% horse serum in PBS. Antibodies against IRS1 (1:50), IRS2 (1:50), and Na/K ATPase (1:100) were used (overnight, 4 °C). Fluorescein-conjugated AffiniPure donkey anti-mouse IgG (1:150) and Cy3-conjugated AffiniPure donkey anti-rabbit IgG (1:250) (Jackson ImmunoResearch laboratories, INC) were diluted in PBS with 3% horse serum and incubated at room temperature for 2 h. Fluorescent images were acquired *via* a Zeiss confocal microscope (Zeiss Confocal LSM 880).

### Surface plasmon resonance

(These experiments were conducted in the Biosensor Core at the University of Maryland School of Medicine). For the determination of C646 bindings to IRβ, IRS1 and 2 proteins, these proteins were immobilized on the surface of a CM5 chip at flow cell 2, 3, and 4, respectively. Binding reactions were done in HBS-P buffer from Biacore (Cytiva Inc), containing 10 mM Hepes, 150 mM NaCl, and 0.05% (v/v) surfactant p20, pH 7.4. This buffer was filtered (0.2 μM) and degassed before use. In order to minimize mass transport effects, the binding analyses were performed at flow rate of 30 μl per minute at 25 °C. The analyte (C646) was dissolved in HBS-P buffer with 0.05% P20 and 2% DMSO and injected into flow cell 1, 2, 3, 4, and the association of analyte and ligands (IRβ, IRS1 and 2 proteins) was recorded respectively by Biacore T200 (Biacore, Inc). Next, the surface was washed with buffer for 600 s to dissociate the analyte–ligand complexes. The signal from the Blank channel (flow cell-1) was served as a control to subtract from the channel containing IRβ, IRS1 and 2 proteins. The binding was removed by injecting 30 μl of glycine (pH 1.5) followed by injection of 30 μl of HBS-P (pH 7.4). For the determination of IRβ binding to IRS1 and 2 proteins, IRβ was immobilized on the surface of a CM5 chip, and the IRS1 or IRS2 protein was injected with different concentrations of C646. Sensorgrams of the interaction were generated by the instrument and analyzed using the software BIAeval 4.0 (Biacore Inc). The reference surface data were subtracted from the reaction surface data to eliminate refractive-index changes of the solution, injection noise, and nonspecific binding to the blank surface. A blank injection with buffer alone was subtracted from the resulting reaction surface data. Data were globally fitted to the Langmuir model for a 1:1 binding.

### Statistical analyses

Statistical significance was calculated with a Student’s *t* test and ANOVA test. Significance was accepted at the level of *p* < 0.05. Sample size (number of mice) was determined on the basis of our previous studies(20,36). At least three samples per group were chosen for statistical meaningful interpretation of results. Statistical significance (*p* < 0.05) was evaluated in GraphPad Prism using one-way ANOVA for multigroup comparisons or using Student’s *t* test for two-group comparison.

## Data availability

All data are contained in this manuscript, and supporting information is available from the author: Ling He (heling@jhmi.edu) upon request.

## Supporting information

This article contains supporting information.

## Conflict of interest

The authors declare that they have no conflict of interest with the contents of this article.
