# Supplementary material for: The P300 acetyltransferase inhibitor C646 promotes membrane translocation of insulin receptor protein substrate and interaction with the insulin receptor
Source: J Biol Chem. 2022 Jan 21;298(3):101621. doi: 10.1016/j.jbc.2022.101621 (PMC8850660; doi:10.1016/j.jbc.2022.101621)
Supplement: Supplemental Figures S1–S4 [file mmc1.pdf]

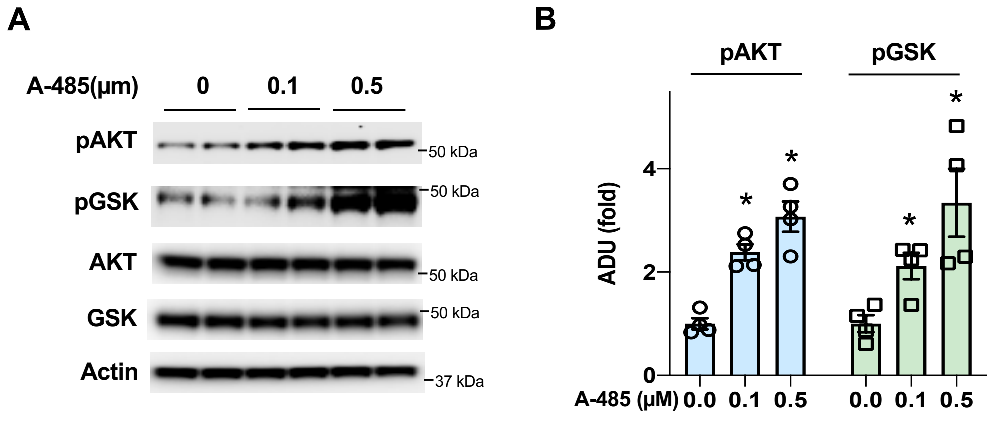

Supplementary Figure 1. ***A*, *B*,** Hepa1-6 cells were cultured in DMEM supplemented with 5% FBS and treated with indicated amounts of A-485 for 3 h (***A***) and densitometric analysis of pAKT and pGSK (***B***) (n=4). *, $p<0.05$, paired sample *t*-test between groups.

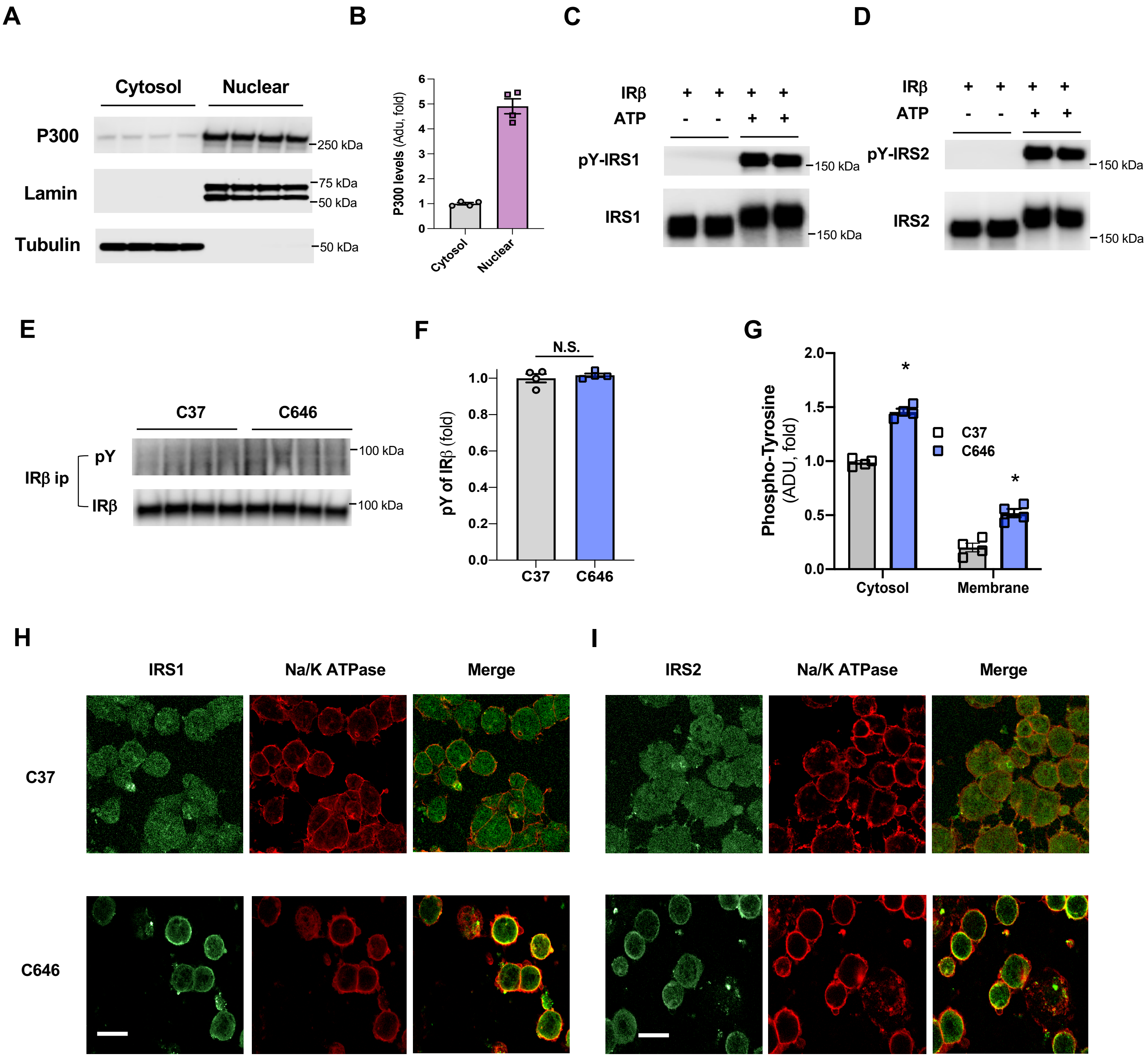

Supplementary Figure 2. ***A***, ***B***, Cytosolic and nuclear fractions were prepared from Hepa1-6 cells using Subcellular Protein Fractionation Kit (Thermo Scientific). 20 µg of protein was loaded (***A***) and densitometric analysis of P300 (***B***). ***C***, ***D***, Purified IRS1 (***C***) and 2 (***D***) proteins were incubated with 0.2 µg of IRβ at 37^{0}C for 1 h in the absence or presence of ATP. Notably, the tyrosine phosphorylation of IRS caused mobility shift in immunoblots. ***E***, ***F***, Hepa1-6 cells were treated as in Fig. 2, cell lysates were immunoprecipitated with IRβ at 4^{0}C overnight, and immunoblotted with anti-phospho-tyrosine antibody (***E***) and densitometric analysis of tyrosine phosphorylation of IRβ (***F***) (n=4). N.S., not significant. ***G***, Densitometric analysis of tyrosine phosphorylation in Fig. 2*E*. ***H***, ***I***, Primary hepatocytes prepared HFD-fed mice were treated as in Fig. 2*I*, *J*. *, p<0.05, paired sample *t*-test between groups. Scale bar, 30 µm.

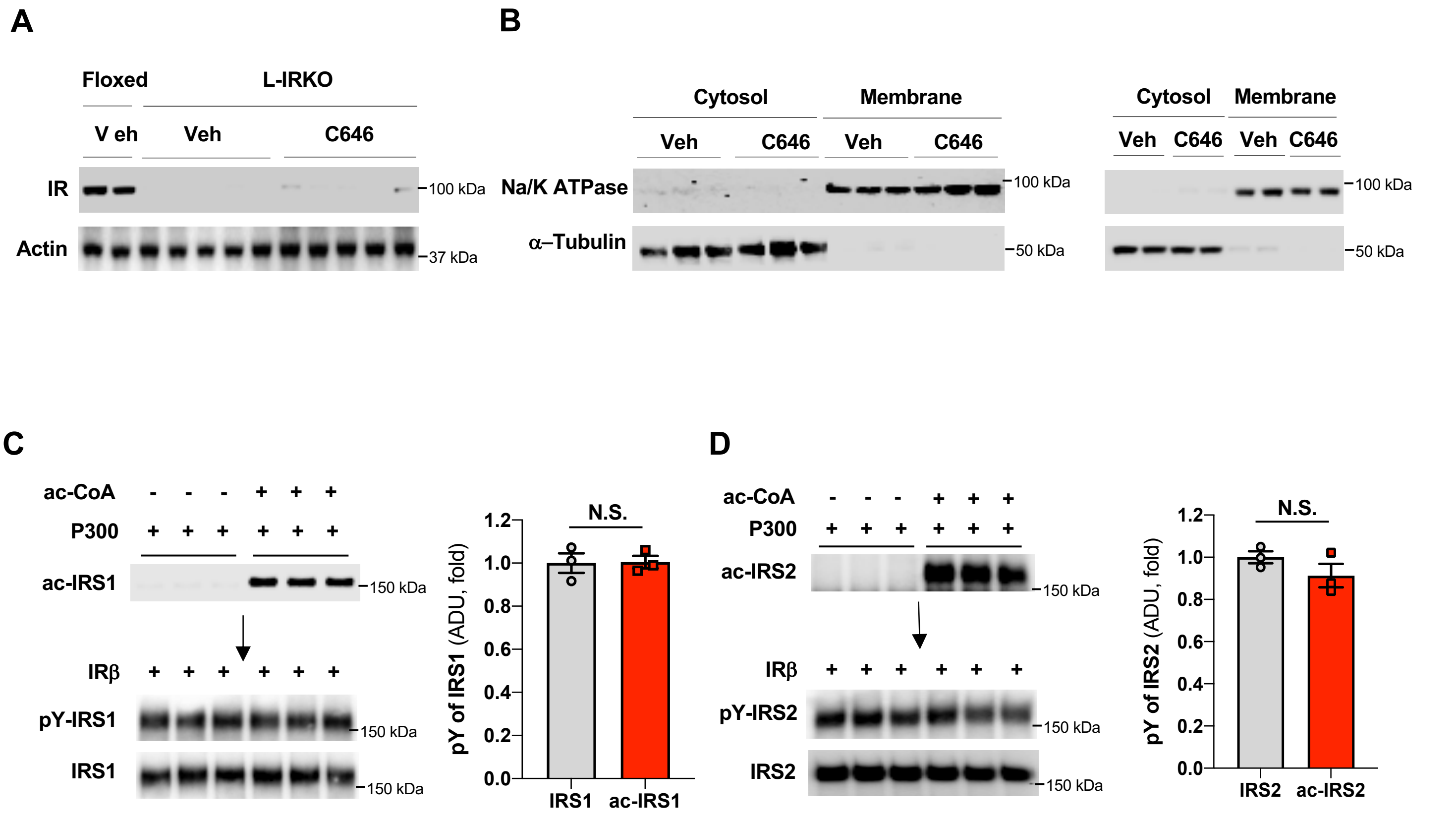

Supplementary Figure 3. ***A*****,** The protein levels of IR in the liver of floxed IR and liver-specific IRKO mice as in Fig. 3*E-J*. ***B***, Cytosolic and membrane fractions were prepared from the liver tissues of mice treated with vehicle or C646. Each lane represents an individual mouse sample (n=5/group). ***C***, ***D***, Purified IRS1 (***C***) and 2 (***D***) proteins were incubated with 0.2 μg of P300 protein in the absence or presence of acetyl-CoA at 37^{0}C for 2 h, then 0.2 μg of IRβ was added (30 min, 30^{0}C). N.S., not significant.

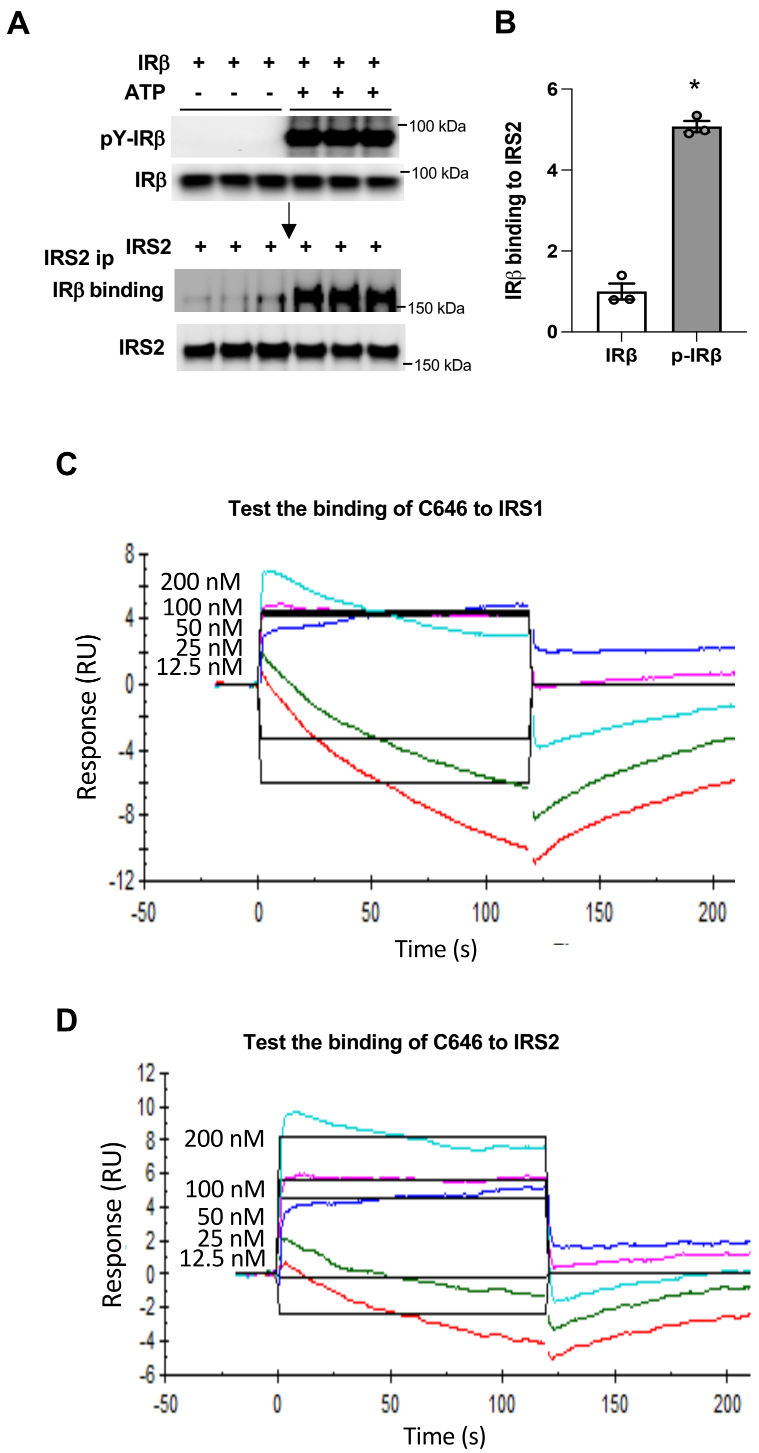

Supplementary Figure 4. ***A***, ***B***, Phosphorylated or unphosphorylated IRβ was incubated with IRS2 for 2 h, then IRS2-specific antibody was used to pull-down IRS2-associated IRβ at 4^{0}C overnight (***A***), and densitometric analysis of IRS2-associated IRβ (***B***) (n=3). ***C***, ***D***, IRS1 (***C***) and IRS2 (***D***) were immobilized on the surface of a sensor chip as with IRβ (Fig. 4*D*), and different concentrations of C646 were injected and the bindings of C646 to IRS were recorded in Biacore T200.
